# Cases of Remitting Fever, with Remarks

**Published:** 1843-12

**Authors:** John Dawson

**Affiliations:** Jamestown, Ohio


Art. II.—*Cases of Remitting Fever, with Remarks.* By John Dawson, M. D., of Jamestown, Ohio.

I propose now, to extract from my Note Book a few cases, as a fair sample of *remitting* fever, as it has occurred in my practice for the last two years.

The cases which I shall give have occurred upon the summit of the table-lands, lying between the Scioto and Miami Rivers. This region, having but few streams of water, and these necessarily very small, is deficient in those annual alluvial deposits, found upon the margins of our larger watercourses, and which are supposed to be so fertile in the production of marsh miasmata. Exemption, however, from malaria is here by no means complete. During the winter and spring, the concavities upon the surface of the country, known as *slashes*, become filled with water. Into these the leaves, limbs, and every species of vegetable trash are washed, where they remain to undergo decomposition during the hot months of summer and early autumn. From this circumstance we frequently have a pretty fair share of intermittent fever, remote from any stream of water.

Case I.—*History*.—Mr. J. P., aet. 33, was taken sick August 25th, 1840. Naturally of good constitution, he remained in good health till within six months previous to his present illness, when he suffered some trouble and inconvenience from bad digestion. For this tonics were prescribed, which gave little or no relief; and he continued to grow weak with an adynamic tendency of the system, until he called for medical assistance.

*Symptoms*.—Unwilling to lie in bed all the time, he would occasionally get up and walk about the house; tongue coated over with a thin white fur, through which the papillæ protruded, the sides and apex red; temperature of the general surface normal; pain in the head and back; pulse from 75 to 80; the former in the morning, the latter in the evening; alimentary canal and urinary organs without any prominent derangement; thirst inconsiderable at first, but gradually increased; appetite impaired, but not entirely suspended.

*Effects of Remedies*.—No derangement of consequence in the circulation, blood-letting was omitted. The patient was first subjected to the operation of an active cathartic, which cleared the first passages, and made things ready for the means by which the malady was to be subdued. I now prescribed the following combination:—℞. Pulv. nitrat. potassæ ʒj., pulv. ipecac., calomel *aa* gr. xii. M.—Divide into six equal parts, and take one powder every three hours. This combination, which Dr. Eberle says he has used in his practice for fifteen years in remitting fever, and with the best results, was continued, with the necessary intervals, until some evidence of ptyalism made its appearance on the gums. With this, subsidence of the fever was confidently expected, for it is asserted in very positive terms by most authors, that such will be the case. Disappointed, however, in this result, and having the evidence now of gastro-enteritis in its incipient stage, the nitrate of potass was left out of the combination, and the calomel and ipecac. still continued as occasion required. Under the use of these the irritation of the bowels grew worse, the pulse rose from 80 to 100, several local inflammations were developed, which were controlled by counter irritation, as they successively made their appearance, and the decline of the patient in flesh and strength was daily observed. Mucilaginous drinks also were freely administered. Convinced that I had the best authority for pursuing this course, under the circumstances of the case, it was persisted in until its efficacy was fairly tested. Frequently I observed, during the course of the malady, that when the system was not under the influence of the medicine, it revived and put on the appearance of amendment; and that when medication was again resumed, the case immediately assumed an unfavorable appearance; and so often were the calomel and ipecac., together with opium and camphor, as the indications seemed to require, resumed, that the patient gave no evidence of convalescence for the period of six weeks from the time that he was taken ill. Nor do I think that he ever would have recovered had the treatment not been changed for that of the purely *physiological.* With this the patient steadily, but not rapidly recovered, for so great was the emaciation, that "skin and bones" are the only terms that will convey any thing like an accurate idea of the condition to which he was reduced.

Case 2d.—*History.*—M. B., æt. 25, a farmer by occupation, thin, spare make, constitution about ordinary, without hereditary predisposition of any kind, so far as I could learn. This patient was taken with the premonitory symptoms of fever, which lasted a week before the chill made its appearance, and after this another week elapsed, during which time he remained about the house, keeping his bed but a small proportion of the twenty-four hours. So little did he complain when in bed or on foot, that the friends were at a loss to appreciate the fact, that there was a person about the house indisposed. His indifference to his own wants, as well as every thing else, in the forming stage of the malady, and for some time time after it had got complete possession, was not peculiar to this case. Most of our cases of autumnal fevers have lately been similarly marked.

*Symptoms.*—Called to this case June 20th, 1841; the countenance and attitude seemed to speak out in favor of little trouble. As soon however as the *tongue* was exposed, I observed a very prominent protrusion of the papillæ through a yellowish white fur; the edges and apex too were of a dark red, and the whole substance rather moist and flabby. The temperature and color of the skin at the commencement and during the course, varied but little from the natural. The functions of the cerebro-spinal axis were somewhat impaired, which, as the malady progressed, became daily more considerable; bowels had been evacuated regularly, though the discharges were scanty and offensive; the urine had the clear limpid appearance of distilled water at first, but at the acme of the malady it became highly colored, with the secretion considerably impaired. Abdomen not distended, nor tender on pressure, in any part, except slightly in the epigastrium, when I first made examination; but as the disorder progressed, general tenderness was imperfectly developed, though not sufficient to make a decided indication for the use of remedies.

*Effects of Remedies.*—The treatment of this case was commenced without any abstraction of blood. With a brisk cathartic the bowels were opened, and immediately after this, *hydrargyrum cum creta*, in fine grain doses, was administered from two to three times a day. This medicine was given to keep up the alvine discharges and subdue the gastro-intestinal irritation indicated by the appearance of the tongue. In conjunction with mucilaginous drinks, it accomplished the design so well, that the patient was kept constantly under its influence as long as the necessity for medication was required. Gum arabic, flax-seed tea, and slippery elm, afford the resources to which we apply, when mucilaginous drinks are required For the last of these articles the results of my practice give a decided preference. I used it freely in this case with the hydrarg. cum creta, as well as in other cases of the same disease. Cold water, in this case, when the patient expressed a preference for it, was freely admitted, as well as in all the cases of fever I have been called upon to treat for the last four years; and I have had no reason, as yet, to regret the practice. Certainly, when the temperature of the mucous surface is exalted so far above that of the normal as to keep the patient repeating his requests for cold water, but little injury can result from the administration of a sufficient quantity to reduce the temperature to the healthy standard; then his thirst will measurably cease. And will not medicines, even calomel, exercise a more favorable impression under such circumstances, than they otherwise could do, upon a surface depraved by a high grade of temperature. Nor have I been troubled with ptyalism as the result of such a course, when some of the preparations of mercury and cold water had been administered together for weeks. But I will digress no farther, and conclude this case by saying, that it terminated in eleven days under the treatment above detailed.

Case 3d.—*History.*—Mrs. H., æt. 21, was taken ill September, 1841. This patient was the wife of a farmer, and as is usual with that class, her habits were of the active kind, with a temperament decidedly sanguine. As in the previous case, the premonitory symptoms of fever had been at work for nearly two weeks previous to the accession of a chill.

*Symptoms.*—The countenance and attitude, on my first visit, were so slightly altered from the natural, that a favorable prognosis seemed self-evident. As soon however as I saw that the tongue was but slightly coated, and red upon its edges and apex, and with the same protrusion of the papillæ, as in the cases before related, I was assured that the stomach and bowels were already the seat of incipient gastro-enteritis. But this the sequel more fully established. The skin was rather dry, and above the temperature of the healthy standard; pulse in the morning at 80, in the evening it sometimes rose as high as 95, always regular; the alimentary canal was very susceptible to purgatives, as was evinced from a small one taken before I was called; urinary organs at the accession of the disease not at all deranged, but when it had run about one half its course, the evacuations of the urine became almost suspended; there was tenderness at the epigastrium, and rather preternatural sensibility over the entire surface of the abdomen.

*Effects of Remedies.*—The patient having taken a mild purgative before I was called, the way was paved for the administration of remedies designed to control the specific lesion. For this purpose the mercury and chalk was administered. At first it seemed to display too much activity, although it was only taken in doses of four grains twice a-day. Modified in this respect by combining each powder with half a grain of opium, its use was continued, with short intermissions, for fifteen days. The intermissions were just long enough to observe, from time to time, what iimpression had been made upon the disease, and what was best calculated to ensure success. In addition, a selected nourishment was allowed, with cold drinks, and infusion of slippery elm. With this management, the patient convalesced on the 18th day.

Remarks.—These cases, as before observed, are about a sample of remittent fever as it has occurred in this region. Were they those of one season alone, they would be less interesting to the practitioner who designs to keep pace with the changes that take place in diseases peculiar to locality. But for the last four years, they have presented about the same characteristics.

In some respects they were similar, while in others, constitution, predisposition, temperament, etc., induced some modification. The reaction in all was of the moderate kind, without that degree of turbulence or tumultuous action, which would indicate the propriety of depletion. Slight remissions in the morning, which were followed by exacerbations in the evening, were common to them all. I had many cases, however, in which no remissions were perceivable. But the phenomena of the tongue was what held the most universal sway. Seldom a case occurred but what gave evidence of gastro-intestinal irritation by the appearance of the tongue. And when the treatment required for such a lesion was instituted, the issue was favorable; when this was not done, the disease was protracted to an indefinite period, as in the first case detailed. While some others did not get well at all, but terminated fatally with the symptoms of follicular ulceration, or dothinenteritis.

These symptoms bear a strong contrast with those that characterised remitting fever in this region, ten or fifteen years ago. Then, as I am informed by many old and intelligent physicians, who have observed that the fevers of this district have undergone a change, the symptoms were decidedly more boisterous and urgent; such as full, tumid countenance, injected conjunctiva, heat of the general surface, tongue heavily loaded with a white or brown fur, aching pains over the whole system, headache, respiration hurried, pulse frequent and full, and in most cases well marked biliary derangement.

With these symptoms before him, the practitioner formerly went to work with the lancet and purgatives, and if he got a fair start, in nine cases out of ten, he would subdue the disease by the end of the fourth or fifth day.

The most popular purgative was that of calomel and jalap, "*ten and ten.*" In many cases the treatment was commenced with an emeto-cathartic, composed of three grains of antimony, and an ounce of Epsom salts. Failing to yield, after being attacked in this manner, the disease was next approached with calomel alone, or in combination with ipecacuanha. This, with such auxiliaries as were required, was continued until the malady subsided, or the mercurial disease was produced. These are the leading features of the practice found most successful in subduing the endemic febrile affections of this region, from its first settlement until the period of which I have before spoken.

Success now requires the pursuance of a different course. There being, as a general remark, no indications for blood-letting, the only pretext that can be set up in favor of it, is fashion. True, the reason that it might operate as a prophylactic of visceral congestions has been often urged; but such a course finds little favor in theory, and none in practice. What facts I have gathered are decidedly in favor of economising the resources of the system, of maintaining the integrity of the vital current, until the necessity for its abstraction is decidedly evinced.

Inflammations, we know, are engrafted frequently upon opposite conditions of the general system. Doubtless they occur with most facility where the diathesis is sthenic. But that their advent is much favored by weakening the powers of the *vis medicatrix naturæ* by the process of blood-letting, is now so well ascertained, that in fever, more liable to complication than any other general disease, the abstractions of blood should be cautiously performed. These remarks are designed to apply to general blood-letting. Of the value of local depletion, and its comparative superiority in the fevers of this region, I am well convinced. Seldom a case occurs but what it is in demand; and the indications decided, and sometimes even peremptory for its adoption.

Coeval with the many improvements characterising the seventeenth century was the introduction of mercury in the cure of disease. Wavering as has frequently been the confidence of the profession in its utility, in the treatment of fevers, it has now, somehow or other, secured a more general use in the treatment of the autumnal fevers in our own country, than any one remedy with which we are acquainted. From some observation, and a good deal of intercourse with the older physicians of this State, I find that the general use that was formerly made of calomel, found some warrant in the character of the fevers. This is especially the case in regard to the bilious remittent and intermittent fever, which obtained when the country was first settled, and for some time afterwards; and, indeed, until the greatest of our sources of malaria have, by the improvements of the country, been destroyed.

That, however, calomel obtained a currency and reputation, to which it never had any just claims, is a position that is now about to be confirmed. The reported cases and essays coming out now in such abundance, upon the "*abuses of mercury*," find their best elucidation in a general desire to limit the application of the medicine to its proper bounds.

Considered as a purgative, it is applicable to all the stages of fever, where purging is required. But to its continuation until ptyalism, or its specific effect is induced, I object, as being a practice the utility of which, after all our experience, has never yet been demonstrated. We have not yet ascertained, *whether the fever subsides because of the superinduction of the murcurial disease, or in consequence of having run its course upon the system.* Consulting all the cases where the efficacy of ptyalism in any degree, has been extolled in the treatment of fever, we find much that creates doubt, whether the issue should be attributed to the specific effects of mercury, or the law of self-limitation in the disease. Besides it has been no unusual phenomena in my practice to see a fever continue after the mercurial disease had been produced, just as it had done before. But the reader might wish to know whether the murcurial influence was perfect. I answer, it was; as much so as could have been desired. This has been witnessed so frequently by the practitioners in this region, in treating the fevers of the present day, that the idea of curing them by the specific effect of mercury, is almost wholly abandoned. But this is not an observation of modern times. In his Commentaries upon the Aphorisms of Boerhaave, Van Swieten incidentally mentions a case of quartan ague that continued its course in the midst of a salivation. With the effects of salivation in intermittent fever, and all those generally denominated idiopathic, I have had but little experience. Most of the cases which have fallen under my observation have been what the older nosologists would call symptomatic, supported and sustained by a primary focus or *foci* of irritation. In these, where the irritation was in the bowels, more or less difficulty occurred in bringing the system under the influence of the mercurial disease, in consequence of the purging qualities of the medicine. Seated however in any other part of the system, this was not so much the case.

A ptyalism cures "by the general and permanent stimulant power, by which it induces and keeps up an action that ultimately supersedes the morbid one." (Hunter.) Antiquated as is this explanation, it has, so far as I am acquainted, never been superseded. The cure then, is produced by the stimulant effects of mercury. Vague, too, as is the explanation, when we consider the latitudinarian sense in which the word *stimulant* is used, it is nevertheless more definite than the term *alterant,* as employed to express the same idea by modern writers. Both terms are evidently deficient, because they are *ambiguous,* and may be made to express modes of action of a very different nature. So entirely *general* do they also appear to be, that if we had no other evidence, this would be sufficient to show, that our knowledge of the mode in which ptyalism cures disease is extremely crude. We shall not extend our remarks here any farther, inasmuch as the *modus operandi* would be a matter of but little importance, provided the propriety of attempting to cure fever by ptyalism be sustained either by theory or practice.

"It rarely happens," says M. Hall, "that fever consists in mere febrile movements of the system. There are usually complications with the general febrile state, of affections of the head, chest, and abdomen; and it has long been disputed whether these affections be primary causes, or secondary effects of the fever; and much that is just has been urged on both sides of the question. It is singular that no such dispute has been raised in regard to a class of fevers which I shall designate the eruptive. Yet it appears to me that the rash and sore throat of scarlatina, and the rash and catarrh of rubeola, and the other complications of these and other febrile diseases, occupy the same rank as the various local affections, whether of function or of anatomy, which we observe so constantly in other fevers." From this, although it is very modest, we conclude that the distinguished Englishman is among those who advocate the doctrine that all fever is symptomatic. To Broussais, more than to any other author, Andral thinks we are indebted for our present enlightened view of the pathology of fever. To seek for the cause of fever in some morbid alteration of some part of the body, to consider fever as a mere symptom of diseased action in some organ, and to direct the treatment against the local affection, and not against the fever which is a mere effect, are parts of the doctrine of Broussais with which Andral was well pleased. "And," says he, "while I agree with Broussais, that every kind of fever may be attributed to a local cause, I think the localization should not be so confined as he has made it."

Various as have been the views, and interminable as has been the discussion of this subject, the opinions of the mass of the profession are still far from being decided. True, the most eminent of the profession in France and England have espoused the side of the symptomatic theory, still there are some things connected with the subject that keep some of us in a state of suspense. No one could doubt for a moment concerning the character of the cases related in this paper. All would agree that from the history, symptoms, and effects of remedies they were symptomatic. No such unanimity, however, could be obtained in regard to the fevers that formerly prevailed in this region; such, for example, as were cured by bark, purging, vomiting, etc. We are aware of the appeals that have been made to morbid anatomy on this score, but so far they are unsatisfactory, and full of ambiguity. Nor can the effects of remedies be invoked with more success. Modified and diversified as is the character of inflammation and irritation from the various organs which may be implicated, we are yet unacquainted with any form of either that can be cured by the preparations of bark. But with this medicine we very certainly subdue intermittents; and I have been informed by reputable practitioners of the Scioto Valley in this State, that their common practice in the bilious fevers of that region at the present time, is to administer quinine freely as soon as they can get a remission. After having tried all ways to subdue bilious fever in that very malarious district, they find the quinine treatment to be the most expeditious. This information I obtained from Drs. Brown and Griswold, of Circleville. Besides positive, negative evidence is not less abundant. Who would think of making himself useful, or sustaining a reputation in the treatment of intermittent and bilious remittent fevers, by trusting to the effects of remedies addressed to an irritation of the alimentary canal, or to an irritation in any other part of the system?

The fact is that formerly most of the fever that prevailed in this region was idiopathic, now it is generally symptomatic; and the prevailing lesion is in the mucous surface of the alimentary canal, gastro-enteritis. About four years ago, a typhoid epidemic fever prevailed here, in every case of which, there was decided evidence of irritation in the mucous membrane of the bowels. Since the prevalence of that epidemic, almost all the diseases seem to be prone to be complicated with a similar anatomical condition. Pneumonia, hepatitis, and synochus fever, are much more liable than formerly to run into gastro-enteritis, and by this means have their course much protracted. Hence the character of our diseases is different now, from what it was in former times. Now they would do very well by which to sustain the doctrines of Broussais. Formerly they would have supported, in an equally decided manner, the views of Dr. Smith.

*June*, 1843.

